# Manganese-Zeolitic Imidazolate Frameworks-90 with High Blood Circulation Stability for MRI-Guided Tumor Therapy

**DOI:** 10.1007/s40820-019-0292-y

**Published:** 2019-07-23

**Authors:** Zhenqi Jiang, Bo Yuan, Nianxiang Qiu, Yinjie Wang, Li Sun, Zhenni Wei, Yanyin Li, Jianjun Zheng, Yinhua Jin, Yong Li, Shiyu Du, Juan Li, Aiguo Wu

**Affiliations:** 1 Cixi Institute of Biomedical Engineering, CAS Key Laboratory of Magnetic Materials and Devices & Key Laboratory of Additive Manufacturing Materials of Zhejiang Province, Ningbo Institute of Materials Technology and Engineering, Chinese Academy of Sciences, Ningbo, 315201 People’s Republic of China; 2 Hwa Mei Hospital, University of Chinese Academy of Sciences, Ningbo, 315010 People’s Republic of China; 3 University of Chinese Academy of Sciences, Beijing, 100049 People’s Republic of China

**Keywords:** Zeolitic imidazolate frameworks-90, Drug delivery, Magnetic resonance imaging, pH-responsive release, Y_1_ receptor ligand

## Abstract

Zeolitic imidazolate frameworks (ZIFs) as smart drug delivery systems with microenvironment-triggered release have attracted much attention for tumor therapy. However, the exploration of ZIFs in biomedicine still encounters many issues, such as inconvenient surface modification, fast drug release during blood circulation, undesired damage to major organs, and severe in vivo toxicity. To address the above issues, we developed an Mn-ZIF-90 nanosystem functionalized with an originally designed active-targeting and pH-responsive magnetic resonance imaging (MRI) Y_1_ receptor ligand [Asn^28^, Pro^30^, Trp^32^]-NPY (25–36) for imaging-guided tumor therapy. After Y_1_ receptor ligand modification, the Mn-ZIF-90 nanosystem exhibited high drug loading, better blood circulation stability, and dual breast cancer cell membrane and mitochondria targetability, further favoring specific microenvironment-triggered tumor therapy. Meanwhile, this nanosystem showed promising T_1_-weighted magnetic resonance imaging contrast in vivo in the tumor sites. Especially, this nanosystem with fast clean-up had almost no obvious toxicity and no damage occurred to the major organs in mice. Therefore, this nanosystem shows potential for use in imaging-guided tumor therapy.

**Electronic supplementary material:**

The online version of this article (10.1007/s40820-019-0292-y) contains supplementary material, which is available to authorized users.

## Highlights

- Manganese-zeolitic imidazolate frameworks (Mn-ZIF-90) with both high drug loading and magnetic resonance imaging (MRI) in vitro and in vivo were prepared.
- The modification of a newly designed pH-protective and active-targeting Y_1_ receptor ligand reduces the drug release during blood circulation and specifically targets the tumor sites, improving therapeutic efficacy in vivo.
- The combination of nano-size Mn-ZIF-90 and the highly specific Y_1_ receptor ligand promotes the specific drug accumulation in tumor sites.

**Electronic supplementary material:**

The online version of this article (10.1007/s40820-019-0292-y) contains supplementary material, which is available to authorized users.

## Graphical abstract

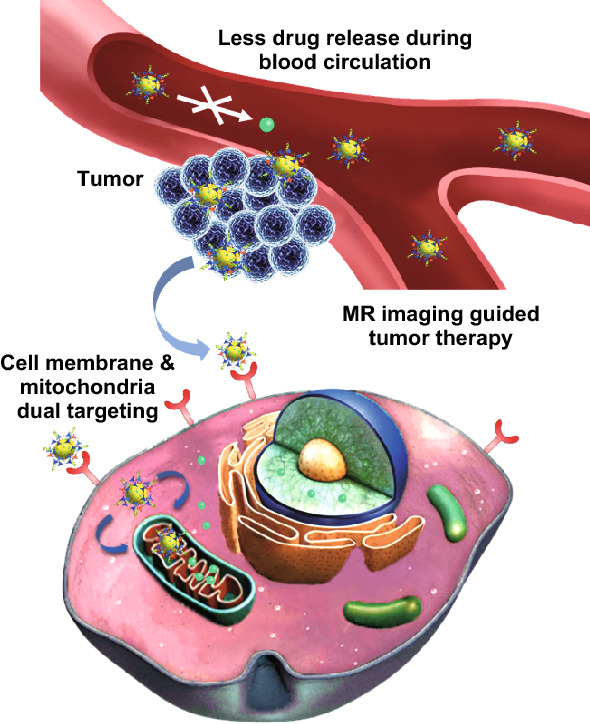

## Introduction

Microenvironment-triggered release plays an important role in controlling the location and concentration of therapeutic drugs in tumor therapy [1–5]. It can be induced by either tumor internal microenvironment or external forces [6], such as pH [7, 8], overexpressed enzyme [9, 10], high concentration of glutathione, temperature [11], microwave [12], and light [13, 14], among others. Among these, the sharp pH gradient between normal tissues and tumorous cellular matrices gives an opportunity for a drug delivery system (DDS) to induce pH-triggered fast drug release at the expected site [10, 15, 16]. In addition, there is an increasing demand for the involvement of imaging components in DDS to track the drug delivery process and provide visible information regarding the change of lesions during therapeutic cycles [7, 17–20]. Therefore, an ideal DDS for tumor therapy should be with high drug loading and itself as an imaging agent should be easily modifiable by various targeting moieties [19, 21–23].

Zeolitic imidazolate frameworks (ZIFs) are thought to be an attractive carrier for both therapeutic drugs and imaging agents due to their high surface areas, tunable pore sizes, and pH-triggered release [24–28]. For example, many therapeutic drugs (DOX or 5-Fu) and imaging agents (IRDye-820 or iron oxide nanoparticles) could be either absorbed or encapsulated into ZIFs by one-pot synthesis. However, many problems still hinder the further application of ZIFs as imaging-guided tumor therapeutic platforms, such as unexpected drug release during blood circulation, serious side effects including death, and low drug loading when carrying both drugs and imaging agents [24, 29]. Although the exchange of Mn to the structure of ZIF-8 could make it suitable for imaging [30, 31], the unexpected toxicity generated from ZIF-8 prevents its further bio-application in vivo [29]. Recently, our previous work and some literature have indicated that ZIF-90, a sister of ZIF-8, showed much lower cytotoxicity than ZIF-8 both in in vitro and in vivo evaluations, and the existence of the aldehyde groups in ZIF-90 makes it easier to modify [32–34]. Furthermore, nanoscale ZIF-90 could release loaded cargoes in mitochondria under high ATP conditions [35], which could improve the therapeutic efficacy of DNA-damaging drugs, because mitochondria play a crucial regulatory role in the intrinsic pathway of apoptosis [36–38]. However, there is still almost no report about the post-synthetic modification of ZIF-90 to make it a magnetic resonance imaging (MRI) contrast agent for both drug delivery and monitoring the expected site in vivo.

**Scheme 1:**
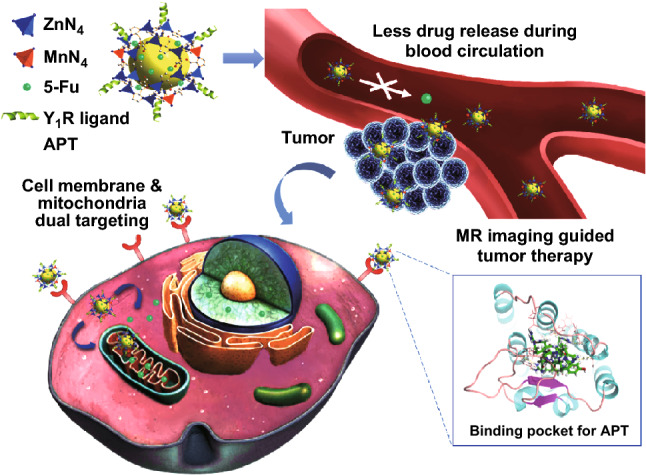
Schematic of bio-application and potential mechanism of APT-Mn-ZIF-90/5-Fu for tumor therapy and the binding mode of APT with Y_1_R. After intravenous injection, the APT-Mn-ZIF-90/5-Fu reduces drug release during blood circulation and triggers fast drug release with low pH and high adenosine triphosphate conditions in the tumor microenvironment for MRI-guided tumor therapy. The binding mode of APT with Y_1_R is shown on the lower right side; Y_1_R is shown in cartoon representation. APT (green carbons) and receptor residues (pink carbons) involved in the ligand binding are shown as sticks. The H-bonds are represented by the yellow dashed lines

Although ZIF nanosystems have been glorified in the past, most of them are still less specific to tumors [24–26]. Peptide ligand modification has been proved to improve the delivery efficiency of DDS with obvious advantages, such as targeting tumor sites, improving hydrophilicity, and prolonging blood circulation [39, 40]. Neuropeptide Y Y_1_ receptor (Y_1_R) is highly overexpressed in human breast tumors and metastases (above 90%), while the normal breast tissues express Y_2_R only [41]. Recently, we found that Y_1_R ligands, such as [Pro^30^, Nle^31^, Bpa^32^, Leu^34^]-NPY(28–36) and [Asn^6^, Pro^34^]-NPY, play important roles in tumor-targeted imaging and therapy with high selectivity to breast tumors and less effect on other organs [39, 42, 43]. Especially, these Y_1_R ligands can reduce premature drug release during blood circulation due to their different spatial configurations under different pH conditions [39]. Therefore, it is of great interest to design novel Y_1_R ligands with both active targeting and pH responsiveness, which might provide a new strategy to solve the problem of the relatively fast drug release of ZIFs during blood circulation and improve the targetability to tumor sites, thereby generating great biological outputs (Scheme 1).

**Fig. 1:**
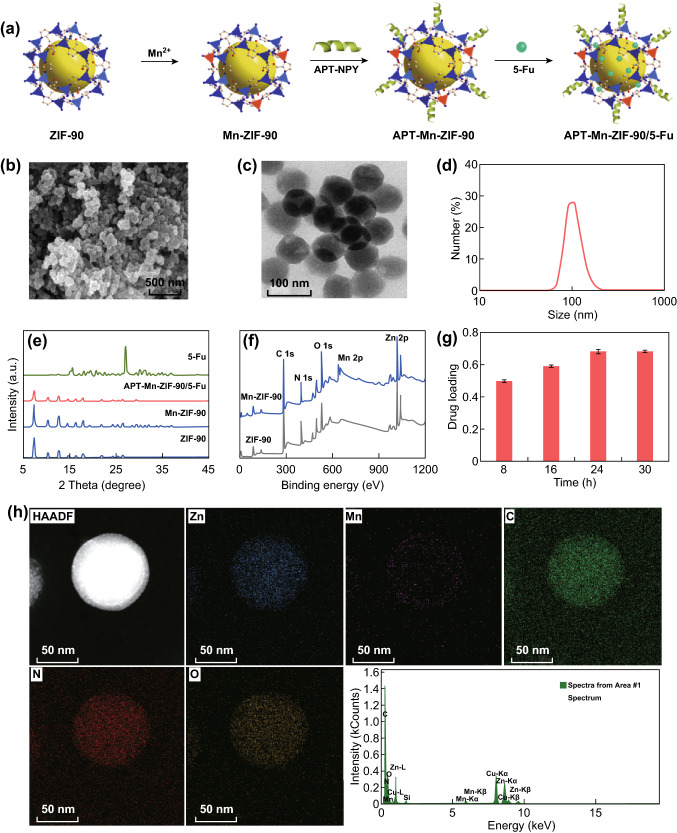
**a** Schematic of APT-Mn-ZIF-90/5-Fu preparation process; **b** SEM and **c** TEM images of Mn-ZIF-90; **d** dynamic light scattering of APT-Mn-ZIF-90 in culture medium at 37 °C; **e** PXRD results of ZIF-90, Mn-ZIF-90, APT-Mn-ZIF-90/5-Fu and 5-Fu; **f** XPS pattern of Mn-ZIF-90 and ZIF-90 NPs; **g** drug-loading content of APT-Mn-ZIF-90 for different stirring times. Mean ± SD (*n *= 3); **h** STEM elemental mapping of Zn, Mn, C, N, O, and EDS spectrum for Mn-ZIF-90

In this work, ZIF-90 was post-synthetically modified by Mn^2+^, then conjugated by an originally designed Y_1_R ligand [Asn^28^, Pro^30^, Trp^32^]-NPY (25–36) (APT) with excellent active-targeting and pH-responsive release (Fig. 1a). The APT-Mn-ZIF-90 showed promising T_1_-weighted imaging both in vitro and in vivo. In addition, a high effective DNA damage drug 5-fluorouracil (5-Fu) was loaded into APT-Mn-ZIF-90 with high drug loading. Meanwhile, APT-Mn-ZIF-90/5-Fu displayed a pH-responsive drug release with low IC_50_ value in vitro and a high blood-drug concentration with effective tumor therapeutic efficiency in vivo. It is more vital to note that the APT-Mn-ZIF-90 showed almost no obvious toxicity and no damage to major organs with fast clean-up in vivo within the dosage that we applied. Therefore, the APT-Mn-ZIF-90/5-Fu with excellent T_1_-MRI contrast could generate efficient therapeutic efficacy with high drug loading, providing an expectation candidate for imaging-guided tumor therapy.

## Experimental

### Preparation of Mn-ZIF-90

The synthesis of ZIF-90 was performed according to pervious reports [24, 35]. Generally, Zn (CH_3_COOH)_2_·2H_2_O was dissolved in 2 mL DMF to 0.2 M and then added to a freshly prepared 2 mL transparent DMF solution of 2-ICA (0.2 M) under vigorous stirring. The product was purified and collected by centrifugation. The product was then dried at 65 °C for 12 h. For the preparation of Mn-ZIF-90, 0.2 mmol ZIF-90 and 0.6 mmol Mn (CH_3_COOH)_2_ were mixed in 10 mL MeOH. Following this, the mixture was transferred to a 25-mL reaction kettle and reacted at 55 °C for 36 h. After the reaction, the product was washed with methanol several times. The solid was soaked in methanol for 4 days, and 20 mL fresh methanol was used to change the solution every 12 h. The product was then collected and dried in vacuum at room temperature overnight and stored at 4 °C.

### Preparation of APT-Mn-ZIF-90 and APT-Mn-ZIF-90/5-Fu

For the preparation of APT-Mn-ZIF-90, the sample was first immersed in methanol for 24 h and then evacuated under vacuum at room temperature for 12 h to remove the solvent of the synthesized Mn-ZIF-90 [30, 44]. 2 mg APT was dissolved with 10 mL methanol in a flask, followed by the addition of 20 mg activated Mn-ZIF-90 nanoparticles (NPs). After another 48 h of stirring at room temperature, the NPs were collected by centrifugation and washed with excess methanol. The residual APT was removed by exchange with methanol before being dried under vacuum.

For the APT-Mn-ZIF-90/5-Fu preparation, 15 mg 5-Fu was dissolved in PBS (pH 7.4) with stirring, and then 5 mg APT-Mn-ZIF-90 was added. The solution was stirred for 24 h in the dark. The samples were collected and freeze-dried. The preparation of Mn-ZIF-90/5-Fu was conducted the same way.

Drug loading = the amount of 5-Fu loaded/the amount of carrier

### Cell Culture

A human breast cancer (MCF-7) cell line was cultured in complete DMEM medium. The medium was supplemented with 10% fetal bovine serum (FBS), 100 units mL^−1^ of penicillin, and 100 mg mL^−1^ of streptomycin. The cells were maintained in a 37 °C incubator with 5% CO_2_.

### Toxicity Evaluation of APT-Mn-ZIF-90 In Vivo

To evaluate the toxicity of APT-Mn-ZIF-90 in vivo, four groups of Balb/c mice were used (*n *= 5), and all the mice were treated with APT-Mn-ZIF-90 (25, 50, or 100 mg kg^−1^) or PBS via a one-time intravenous injection. The survival rate and body weight were recorded for 30 days. To advance our understanding of the toxicity in vivo, another group of treated mice was killed on the 7th day. The collected blood was analyzed by a blood analyzer (Sysmex XT-1800i, Japan), and Hitachi 7600 − 110 autoanalyzer (Hitachi, Tokyo, Japan), and the major organs were collected and stained with Hematoxylin and Eosin (H&E). Furthermore, to investigate the metabolism of APT-Mn-ZIF-90 in healthy mice, each mouse was injected with 100 μL of APT-Mn-ZIF-90 (50 mg kg^−1^) and was killed on days 1, 7, and 14. The main organs were removed, and their Mn concentrations were tested with ICP-OES.

### Drug Release In Vitro

In vitro drug release was performed through a previously reported dynamic dialysis method. For example, 1 mL of Mn-ZIF-90/5-Fu or APT-Mn-ZIF-90/5-Fu (5-Fu: 1 mg mL^−1^) in PBS (pH 7.4) was added to a dialysis bag (MWCO: 2 kDa) and incubated with 49 mL of PBS containing 10% FBS at pH 7.4 or 5.5 in an oscillation incubator at 37 °C with 100 rpm. At predetermined time intervals, some aliquots were removed for analysis and replaced with the same volume of release medium. The drug concentration was determined by UV–Vis spectroscopy.

### Establishment of MCF-7 Breast Tumor Xenograft

This was conducted under the approval of the Regional Ethics Committee for Animal Experiments at Ningbo University, China (Permit No. SYXK (Zhe) 2019-0005). All mice used in this study were bought from Kawensi Biological products sales center (Nanjing, China). Female Balb/c nude mice (18–20 g, 4–6 weeks old) were used to establish the tumor model by a subcutaneous injection of MCF-7 cells (1 × 10^7^ cells).

### MRI In Vivo and Biodistribution of Mn

To investigate the MRI performance in vivo, the tumor-bearing nude mice were intravenously injected with 100 μL of Mn-ZIF-90 and APT-Mn-ZIF-90 (50 mg kg^−1^, [Mn] = 2 mg kg^−1^); then the T_1_-weighted images were acquired using a 1.5 T human clinical scanner (Ingenia 1.5 T, Philips, the Netherlands).

To evaluate the biodistribution of Mn in vivo, four groups of tumor nude mice (*n *= 3) were injected with 100 μL PBS, Mn-ZIF-90, and APT-Mn-ZIF-90 (50 mg kg^−1^). After 1 day, all the mice were killed, then the major organs and tumors were collected, and the distribution of Mn was determined by ICP-OES analysis. For ICP-OES analysis, the organs were freeze-dried and weighed. All organs were treated in aqua regia for 4 h at 95 °C for dissolution of the tissues.

### Pharmacokinetics

For the pharmacokinetics study, the mice were treated with 5-Fu, Mn-ZIF-90/5-Fu, and APT-Mn-ZIF-90/5-Fu (5-Fu: 8 mg kg^−1^) by intravenous injection, and the blood samples (500 μL) were collected at different times. The plasma was collected by centrifugation and extracted with chloroform/methanol (4:1, *v/v*) containing 1% formic acid. The concentrations of 5-Fu in the plasma were tested by HPLC (Agilent, 1260, USA).

### In Vivo Antitumor Activity of APT-Mn-ZIF-90/5-Fu

When the tumor grew to 40–60 mm^3^, 24 mice were divided into four groups. The tumor-bearing nude mice were treated with PBS, 5-Fu, Mn-ZIF-90/5-Fu, and APT-Mn-ZIF-90/5-Fu (100 μL, 5-Fu: 8 mg kg^−1^) via a tail vein injection. The 5-Fu-loaded NPs were injected on days 0, 2, 4, and 6. The tumor volumes and body weights were measured at 2-day intervals. After the treatment, the survival of the mice was observed for 60 days. Furthermore, the major organs and tumors were stained with H&E.

## Results and Discussion

### Characterization of APT-Mn-ZIF-90/5-Fu

As the exchange ratio of Mn^2+^ to Mn-ZIF-90 mainly affects the efficiency of MRI and injection dose, the limitation concentration of Mn^2+^ in Mn-ZIF-90 should first be explored. To prepare Mn-ZIF-90, various synthetic conditions were tested to increase the exchange ratio, such as reaction temperature, time, and molar ratio of Mn^2+^ to ZIF-90 (Figs. S1–S3 and Table S1). When the reaction temperature was increased to over 65 °C, no product could be obtained after centrifugation. Under all the tested conditions, the highest exchange ratio and product yield was achieved when the molar ratio of Mn^2+^ to ZIF-90 was 3 at 55 °C for 36 h. In the product, the ratio of Mn^2+^ to Zn^2+^ was 1:7. Figure 1b, c shows the SEM and TEM images of Mn-ZIF-90; there were no obvious changes in size and shape compared to ZIF-90 (Fig. S4), and the original crystal shape remained distinct. The result of the STEM mapping shows a successful and random exchange of Mn to the ZIF-90 structure (Figs. 1h and S5). The Mn-ZIF-90 had a size around 75 nm and shape similar to ZIF-90. The size of APT-Mn-ZIF-90 in the cell culture medium was around 105 nm, and the zeta-potential was − 2.65 mV (Figs. 1d and S6). The PXRD (Fig. 1e) shows that the Mn-ZIF-90 remained crystalline and iso-reticular to the parent ZIF-90 with SOD topology. The XPS results (Figs. 1f and S7) show that the peak of Mn was exchanged to the structure, and the peaks of Mn 2p were observed at 639.8 (Mn 2p_3/2_) and 652.7 eV (Mn 2p_1/2_) after reaction, which also indicates the exchange of Mn^2+^ to Zn^2+^. In addition, FT-IR spectroscopy indicates that the APT was linked to the surface of Mn-ZIF-90, according to the new peak at 1590 cm^−1^ (C=N) (Fig. S8).

The drug-loading content was highly dependent on both the stirring time and the mass ratio of 5-Fu to ZIFs (Figs. 1g, S9, and S10). The drug concentrations of Mn-ZIF-90 and APT-Mn-ZIF-90 increased with time within 24 h, and there was no obvious increase later. The highest drug loading was found when the mass ratio of 5-Fu to ZIFs was 1:3 for 24 h. Under the same condition, the drug-loading content of APT-Mn-ZIF-90 was 0.6793 g/g 5-Fu, which was a little lower than that for Mn-ZIF-90 (0.6942 g/g), but still higher than for ZIF-90 (0.6204 g/g) (Fig. S11) and those of previous reports [24, 33, 45]. The high drug-loading content might be attributed to the specific adsorption between the 5-Fu six-membered ring and imidazolate, or even the electrostatic adsorption and increased N_2_ absorbance after modification (Fig. S12). Further, PXRD showed no characteristic peak of 5-Fu in APT-Mn-ZIF-90/5-Fu, indicating that the 5-Fu was un-crystallized in APT-Mn-ZIF-90; the un-crystallized small molecule drugs would induce cancer cell death more effectively [24, 46].

### Cellular Uptake and Active Targetability of APT-Mn-ZIF-90/5-Fu In Vitro

**Fig. 2:**
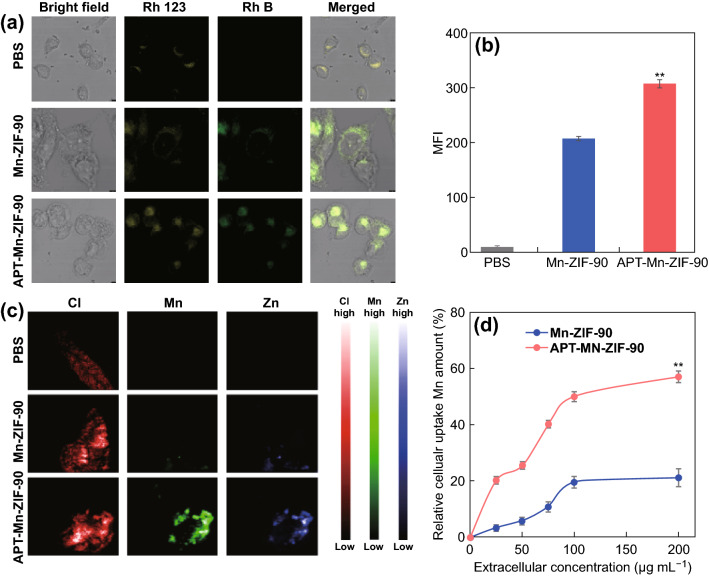
Cellular uptake of NPs in MCF-7 cells. **a** LSCM images of RhB-loaded Mn-ZIF-90 and APT-Mn-ZIF-90 incubated with MCF-7 cells for 8 h, and the RhB 123 was used as a mitochondrion tracker; **b** mean fluorescence intensity (MFI) of MCF-7 cells incubated with NPs for 8 h, and followed by flow cytometric analysis; **c** XRFM images of Cl, Zn, and Mn in MCF-7 cells incubated with NPs for 8 h; **d** the concentration of Mn^2+^ in MCF-7 cells after incubating with different concentrations of NPs for 24 h. Mean ± SD (*n *= 3), **p < 0.01

The distribution of nanoparticles in the tumor cells was investigated using a laser scanning confocal microscope (LSCM). Fluorescent dye rhodamine B (RhB) was encapsulated into Mn-ZIF-90 and APT-Mn-ZIF-90 to track their cellular uptake. After incubating with human breast cancer cell line MCF-7 for 8 h, the RhB showed strong fluorescence in the intracellular compartment. Further, the mitochondria of the MCF-7 cells were stained with rhodamine 123 [35], and the fluorescence of RhB showed a good correspondence with rhodamine 123 (Fig. 2a), indicating the RhB was released in the mitochondria. Therefore, once the RhB was replaced by 5-Fu, the drugs could also be released in the mitochondria, and the DNA-damaging drugs would show a better efficacy by damaging the mitochondria DNA that is hard to be repaired [38, 47]. To further characterize the distribution of elements, X-ray fluorescence microscopy (XRFM) was used to locate the distribution of Zn and Mn. As shown in Fig. 2c, the intensity of Zn and Mn was basically the same everywhere, which also indicates that the NPs were integrated into the tumor cells and their subcellular organelles. Similar results were also observed in the soft X-ray fiber microscopy, which showed that the NPs were in the edges of the cells (Fig. S13).

To determine the active targetability of APT-Mn-ZIF-90 (Fig. 2b), the RhB was loaded inside the APT-Mn-ZIF-90 and analyzed by flow cytometry. The mean fluorescence intensity (MFI) of APT-Mn-ZIF-90 (307.4) was approximately 1.5-fold higher than that of Mn-ZIF-90 (207) after 8 h of incubation. The same result was also found in the element mapping, in which the APT-Mn-ZIF-90 group showed stronger intensity. In addition, the MCF-7 cells were incubated with different concentrations of NPs for 24 h, and Mn was tested as a marker (Fig. 2d). With the increasing NP concentration, the relative endocytosis ratio also increased. The APT-Mn-ZIF-90 groups showed a higher ratio at all tested concentrations. According to a previous report, simultaneously targeting the cell membrane and mitochondria is an effective way to increase therapeutic efficacy of DNA-damaging drugs [38]. The above results show that the modification of APT could increase the concentration of NPs inside tumor cells; therefore, the nanoparticles might further release the drugs in the mitochondria, increasing the therapeutic efficacy of 5-Fu.

To reveal the active-targeting mechanism of APT, the binding mode of APT to Y_1_R was investigated. As shown in Fig. S16, the interaction between APT and Y_1_R was dominated by the formation of the H-bonds. Specifically, Arg1, Asn5, Arg9, and Gln10 in APT formed intermolecular H-bonds with the backbones of residues Asn186, Cys198, Phe282, and Phe286 in the Y_1_R, respectively. The side chain of Asp31, Tyr100, and Asn283 in the Y_1_R formed H-bonds with the residue Arg1, Asn5, and Tyr12 in the APT. In addition, there was also a π-π interaction between the residue Try8 of the APT and the residue His306 of the Y_1_R. Moreover, a salt bridge interaction existed between the residue Arg1 of APT and the residue of Asp104 and Asp31 in Y_1_R. The main binding interface of APT to Y_1_R was similar to that in a previous report [48], which was mainly dominated by H-bond, π-π stacking, salt bridge, and hydrophobic interactions. APT underwent great conformational changes to enable the side chains to occupy the binding pocket of Y_1_R. In addition, the binding energy of APT calculated by a molecular dynamics simulation was − 1320 kJ mol^−1^, indicating that APT displayed strong interaction with Y_1_R, compared to our previous report [49].

### Drug Release and Cell Inhibition Effect of APT-Mn-ZIF-90/5-Fu In Vitro

**Fig. 3:**
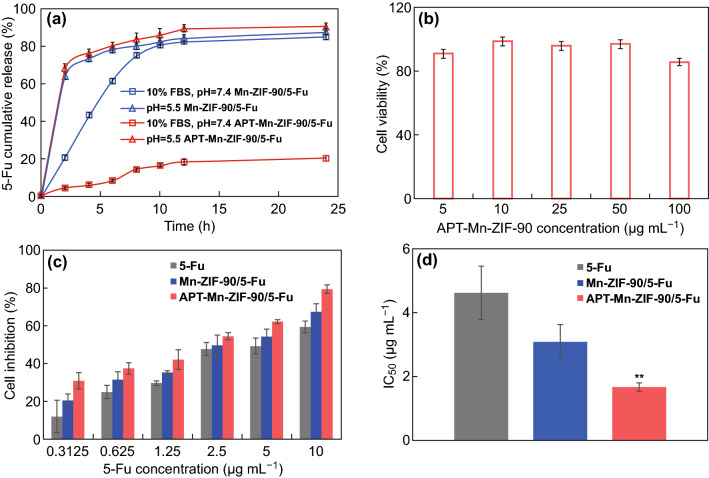
**a** Cumulative release of 5-Fu from NPs in different environments (pH 7.4 + 10% FBS and pH 5.5) for 24 h in 37 °C in vitro; **b** cell viability of APT-Mn-ZIF-90 incubated with MCF-7 cells for 24 h; **c** cell inhibition effect and **d** IC_50_ values of 5-Fu, Mn-ZIF-90/5-Fu, and APT-Mn-ZIF-90/5-Fu after incubation with MCF-cells for 24 h. Mean ± SD (*n *= 3), **p < 0.01

Before testing the cell inhibition effect of the NPs, in vitro drug release was first investigated. As shown in Fig. 3a, approximately 60% of 5-Fu was released from Mn-ZIF-90 at pH 5.5 within 2 h; the same trend could also be seen in the APT-Mn-ZIF-90 group. The fast drug release from DDS in the tumor sites within a short time is a key factor to improve the drug concentration in tumor cells and further improve its therapeutic efficacy. Meanwhile, the release of Mn-ZIF-90 and APT-Mn-ZIF-90 was also tested under the condition of pH 7.4 with 10% FBS, which was used to simulate the human blood environment. It showed that approximately 60% and 80% of 5-Fu were released from Mn-ZIF-90 at 6 and 24 h, respectively. However, only approximately 20% of 5-Fu was released from APT-Mn-ZIF-90 within 24 h. This result indicates that the modification of APT could decrease the drug release at physiological pH level during blood circulation; therefore, more of the drug could be accumulated in the tumor site [47]. Moreover, the reduced drug release before reaching the tumor site could also minimize the side effects on major organs caused by an untargeted free drug [11].

Afterward, the cytotoxicity of APT-Mn-ZIF-90 was evaluated. The cell viabilities of the MCF-7 cells were all over 80% at the tested concentrations from 5 to 100 μg mL^−1^ after incubation with APT-Mn-ZIF-90 for 24 h (Fig. 3b), indicating a good biocompatibility of APT-Mn-ZIF-90. To determine the cell inhibition effect of drug-loaded NPs, different concentrations of 5-Fu, Mn-ZIF-90/5-Fu, and APT-Mn-ZIF-90/5-Fu were incubated with the MCF-7 cells for 24 h at an equivalent concentration of 5-Fu. As a result of the CCK-8 test, Fig. 3c shows that the Mn-ZIF-90/5-Fu demonstrated a higher cell inhibition than the free 5-Fu. This increase can be attributed to the 5-Fu release from Mn-ZIF-90 in the mitochondria, where further DNA damage was induced. A previous report indicates that the DNA in the mitochondria is unable to repair itself and the injured mitochondria, resulting in the apoptosis of cells more easily. Besides this, the modification of APT increased the drug concentration in tumor cells, causing a much lower IC_50_ value (1.643 μg mL^−1^) than those of 5-Fu (3.07 μg mL^−1^) and Mn-ZIF-90/5-Fu (4.6 μg mL^−1^) (Fig. 3d) (*p *< 0.01). Therefore, simultaneous cell membrane and mitochondria active targeting can increase the drug concentration in tumor cells and damage DNA in mitochondria, causing the cell apoptosis more easily [49].

### Biocompatibility and Biodistribution of APT-Mn-ZIF-90/5-Fu In Vivo

**Fig. 4:**
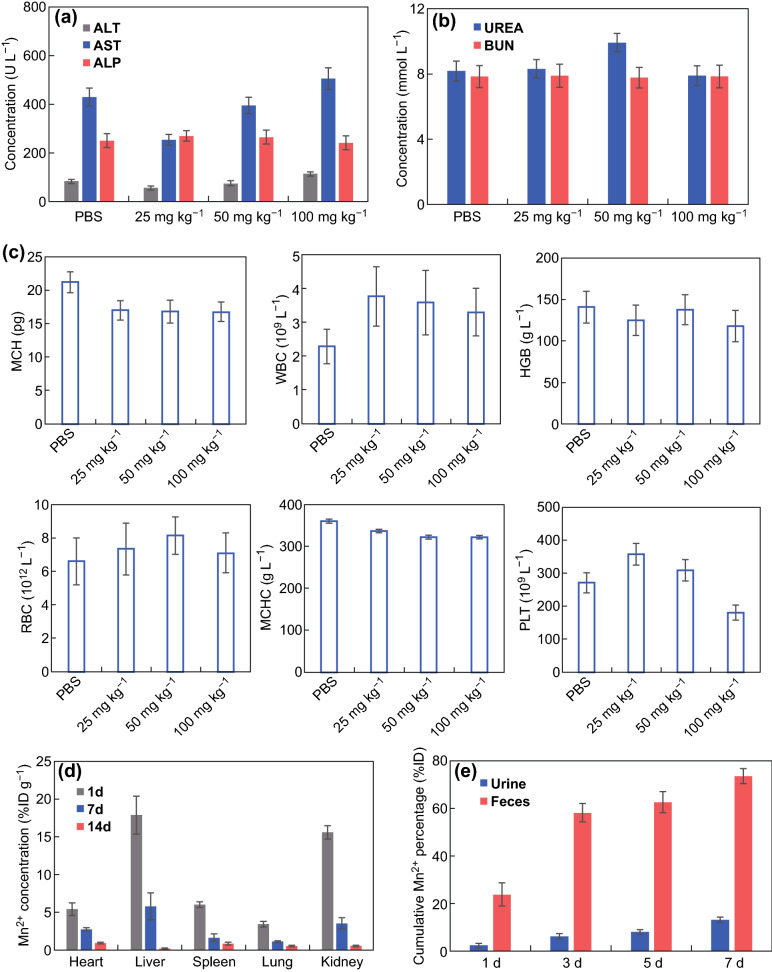
**a**, **b** Serum biochemistry data of healthy mice after intravenous injection of APT-Mn-ZIF-90 for 7 days at different concentrations; **c** blood routine analysis. Blood levels of MCH, WBC, HGB, RBC, MCHC, and PLT of healthy mice after APT-Mn-ZIF-90 injection after 7 days at different concentrations; **d** biodistribution and **e** excretion profiles of Mn^2+^ after treatment with APT-Mn-ZIF-90 at different times. Mean ± SD (*n *= 3)

**Fig. 5:**
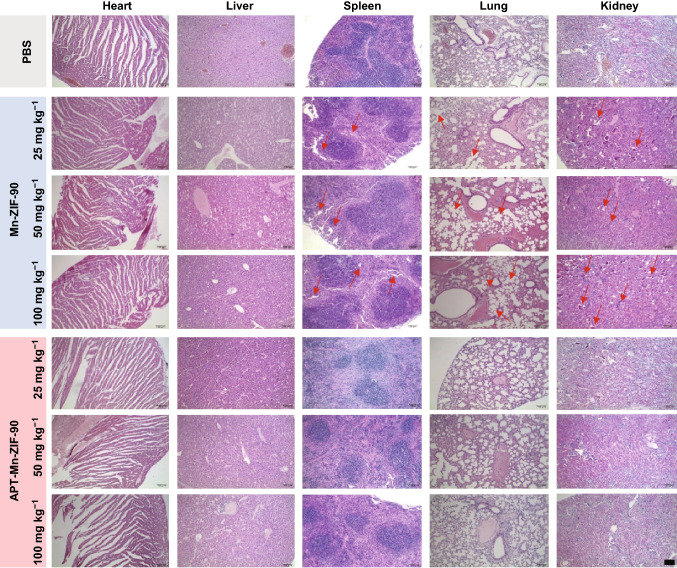
H&E staining of major organs of mice 7 days post-injection of PBS and different concentrations of Mn-ZIF-90 or APT-Mn-ZIF-90. (Scale bar = 100 μm)

Before the bio-application of Mn-ZIF-90 in vivo, its biocompatibility was carefully evaluated [50]. To conduct the in vivo toxicity evaluation of Mn-ZIF-90 and APT-Mn-ZIF-90, different concentrations (12.5–100 mg kg^−1^) of NPs were injected into the mice via the tail vein. As a result, there was no death, abnormal behavior, or obvious weight decrease over the 30 days post-injection (Table S2 and Fig. S17). To further understand the effect of APT-Mn-ZIF-90 on the blood and major organs, another group of mice administered with different concentrations of NPs were killed on day 7, and blood was collected for blood routine and biochemical analyses (Fig. 4a–c). There was no obvious decrease in the white blood cell (WBC) and red blood cell (RBC) counts. Although platelet (PLT) count decreased at the concentration of 100 mg kg^−1^ compared to that in the PBS group, it was still in the normal range at 50 mg kg^−1^ [49]. Furthermore, there was no apparent difference in the ratio of AST to ALT between all groups, indicating that no extensive damage to the liver occurred. As shown in the H&E staining images (Fig. 5), some marked damage can be observed to the spleen, lung, and kidney for all the tested concentrations of Mn-ZIF-90 after 7 days, but the APT-Mn-ZIF-90 group showed no obvious damage in all tested major organs, even at 100 mg kg^−1^. These results indicate that the modification of APT minimized the damage of Mn-ZIF-90 to major organs.

To further evaluate the excretion kinetics of the NPs in vivo, the Balb/c mice were killed on days 1, 7, and 14 after the intravenous injection of APT-Mn-ZIF-90, and the concentration of Mn^2+^ in various organs was analyzed by ICP-OES (Fig. 4d). Mn^2+^ was approximately 18% and 15% ID g^−1^ in the liver and kidney, respectively, on day 1, which reduced to less than 1% ID g^−1^ after 14 days. Compared to most of the traditional inorganic NPs, Mn^2+^ could be cleared within 14 days. Further, the organ coefficient of the major organs did not show a significant increase compared to that in the PBS group during the 14 days of observation, which also indicates that APT-Mn-ZIF-90 was cleared within 14 days (Fig. S18). The Mn^2+^ concentration in the urine and feces was also tested by ICP-OES (Fig. 4e). Via feces, 20% ID cumulative Mn^2+^ was cleared within 1 day. In addition, approximately 70% and 15% ID cumulative Mn^2+^ was detected in the feces and urine, respectively, after 7 days, indicating that most of the NP_S_ could be cleared within 7 days. Due to the noncovalent coordination interaction of ZIFs, APT-Mn-ZIF-90 can be decomposed much easier than the other inorganic materials [24], and the metal ion and organic ligand can be cleared fast [51]. The above results suggest that APT-Mn-ZIF-90 had low toxicity and was cleared faster in vivo.

### In Vitro and In Vivo MRI and Biodistribution of APT-Mn-ZIF-90/5-Fu in Tumor-Bearing Mice

**Fig. 6:**
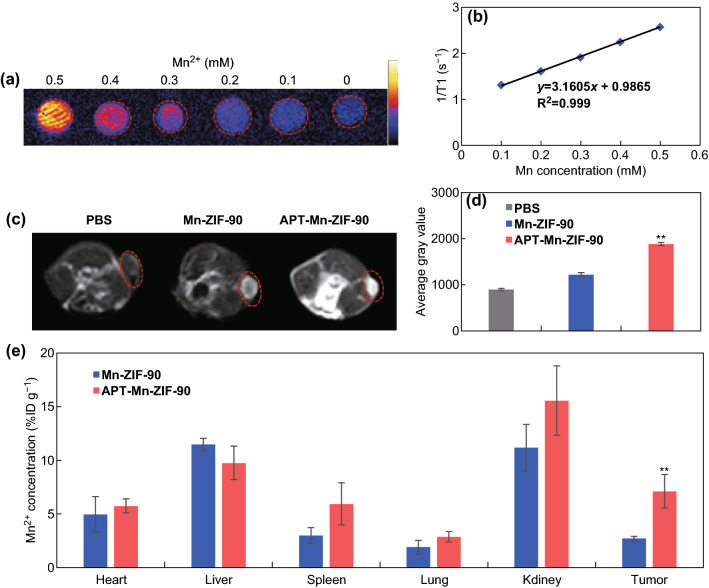
**a** In vitro T_1_-weighted MR images of APT-Mn-ZIF-90 at different concentrations; **b** T_1_ relaxation rate of APT-Mn-ZIF-90; **c** T_1_-weighted MR images of MCF-7 tumor-bearing nude mice after intravenous injection of PBS, Mn-ZIF-90, and APT-Mn-ZIF-90 at 24 h, the tumor is marked by the red circle. **d** Average gray value of tumor from the T_1_-weighted images; **e** biodistribution of Mn in major organs and tumor after injection for 24 h. Mean ± SD (*n *= 3), **p < 0.01

Due to the five unpaired 3d electrons of Mn^2+^, Mn-based NPs can be used as effective T_1_ contrast agents in MRI [52]. APT-Mn-ZIF-90 showed a concentration-dependent brightening contrast effect in the T_1_-weighted imaging in vitro (Fig. 6a). As shown in Figs. 6b and S19, the *r*_1_ value of APT-Mn-ZIF-90 was 3.160 mM^−1^/s, and the *r*_2_/*r*_1_ value was 1.802, indicating the suitability of the NPs for T_1_-weighted imaging. After the intravenous injection of APT-Mn-ZIF-90 and Mn-ZIF-90 into the tumor-bearing mice for 24 h (Fig. 6c), T_1_-weighted MR signals could be observed from the tumor site, compared to the PBS group. The average gray value of APT-Mn-ZIF-90 (1881.16) was higher than that of Mn-ZIF-90 (1217.63), indicating that more NPs were accumulated in the tumor after the modification of APT (Fig. 6d).

To evaluate the biodistribution of APT-Mn-ZIF-90 in vivo, the main organs and tumors of the mice were collected 24 h post-injection, and the concentration of Mn^2+^ was determined by ICP-OES (Fig. 6e). There was no obvious accumulation of Mn^2+^ in the major organs after the modification of APT. The APT-Mn-ZIF-90 (7.142% ID/g) showed a much higher tumor accumulation than Mn-ZIF-90 (2.763% ID/g), which was consistent with the MRI results. This higher accumulation of NPs could be attributed to either the modified APT generating a better active targetability in vivo, or probably a longer blood circulation time.

### In Vivo Pharmacokinetics and Antitumor Efficacy of APT-Mn-ZIF-90/5-Fu

**Fig. 7:**
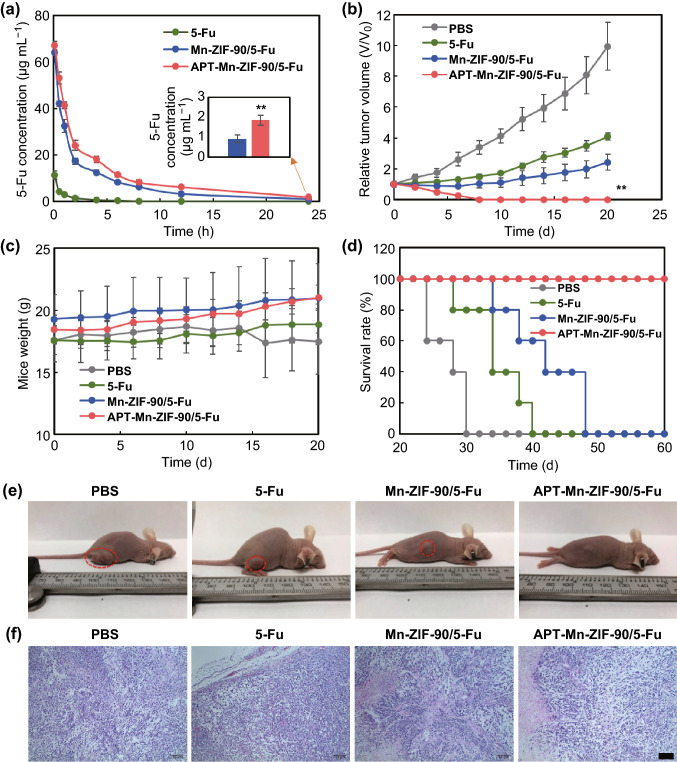
**a** Blood concentration of 5-Fu at different time points after intravenous injection of 5-Fu, Mn-ZIF-90/5-Fu, and APT-Mn-ZIF-90/5-Fu, with 5-Fu concentration of 8 mg kg^−1^. The pharmacokinetics of 5-Fu follows the three-compartment model. Mean ± SD (*n *= 3); **b** relative tumor volume, **c** body weight, and **d** survival rate of tumor-bearing mice after treatment with PBS, 5-Fu, Mn-ZIF-90/5-Fu, and APT-Mn-ZIF-90/5-Fu, with 5-Fu concentration of 8 mg kg^−1^. Mean ± SD (*n *= 5), **p < 0.01; **e** photograph of tumor-bearing mice 14 days post-injection; **f** H&E staining of tumors for one treatment after 24 h (scale bar = 100 μm)

Encouraged by the good performance of APT-Mn-ZIF-90/5-Fu in drug release and cell inhibition in vitro, its pharmacokinetics and antitumor effects were evaluated in vivo. The blood concentration of 5-Fu decreased over time for the three tested groups after intravenously injecting an equivalent 5-Fu of 8 mg kg^−1^ (Fig. 7a). Furthermore, no 5-Fu was observed after 8 h for the free 5-Fu group, but 5-Fu in the other two groups remained at a relatively high concentration after 24 h. The blood circulation half-lives (t_1/2_) were 6.8 and 4.0 h for APT-Mn-ZIF-90 and Mn-ZIF-90, respectively, which were 5.1- and 3.0-folds higher than that of free 5-Fu. At 24 h, the blood concentration of APT-Mn-ZIF-90/5-Fu (1.842 μg mL^−1^) remained two times higher than that of the Mn-ZIF-90/5-Fu group (0.9148 μg mL^−1^). The higher drug concentration and long t_1/2_ can be attributable to fact that the active-targeting APT generated pH-protective release during blood circulation.

The in vivo antitumor efficacy was tested for different groups after four administrations. The tumor sizes of the treated groups were monitored for 20 days (Fig. 7b). It was exciting to observe that the tumor grew slowly during the administration process of APT-Mn-ZIF-90/5-Fu, and the tumor was eliminated, with no recurrence, within 20 days (Fig. 7e). Meanwhile, the tumors of the other three groups all grew quickly over the observation time. The H&E staining images (Fig. 7f) show that there were neuromas of tumor vacuole for the APT-Mn-ZIF-90/5-Fu group compared to the other groups. It is worth noting that all the mice treated with APT-Mn-ZIF-90/5-Fu showed no obvious tumor recurrence and survived for more than 60 days, while the average life spans with PBS, 5-Fu, and Mn-ZIF-90/5-Fu were 26, 35, and 44 days, respectively (Fig. 7d). The aforementioned excellent therapeutic efficacy and prolonged survival might be due to the following two reasons: first, the longer blood circulation time and higher drug blood concentrations led to higher drug concentration in tumors; second, the simultaneous cell membrane and subcellular targeting increased drug concentration in tumor cells and induced the cell apoptosis.

**Fig. 8:**
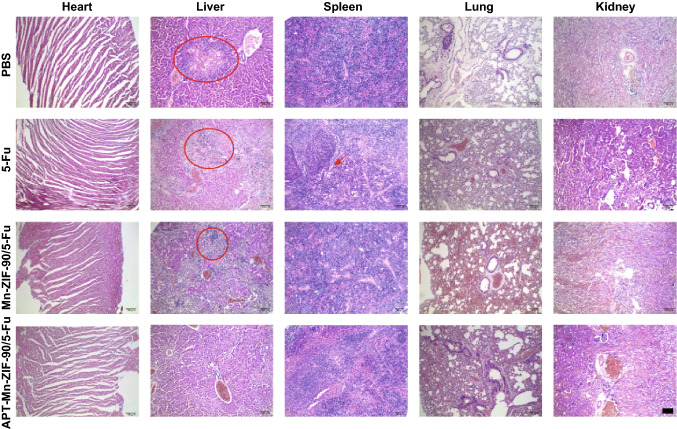
H&E staining of major organs of MCF-7 tumor-bearing mice after treatment for 14 days. The metastasis are marked by the red cycle in the liver (scale bar = 100 μm)

Furthermore, there was no obvious body weight decrease after the treatment with APT-Mn-ZIF-90/5-Fu for 20 days (Fig. 7c). However, the PBS group showed a decrease in body weight from day 16. Additionally, tumor metastasis was found in the livers of the PBS group from the H&E staining images (Fig. 8), which may be attributed to the decrease in body weight, even causing the death. The same tumor metastasis could also be found in the 5-Fu and Mn-ZIF-90/5-Fu groups, and not the APT-Mn-ZIF-90/5-Fu group. In addition, some organ damage was found to the spleen treated with 5-Fu, but could not be found in the APT-Mn-ZIF-90/5-Fu group. These results indicate that the APT-Mn-ZIF-90 could depress tumor metastasis and decrease the side effects of 5-Fu on the major organs after therapy. More specifically, APT-Mn-ZIF-90/5-Fu showed low toxicity with fast clean-up and efficient antitumor efficacy with accompanying excellent MRI performance in vivo, which can be expected to be an efficient nanoplatform in tumor precision medicine.

## Conclusions

In this work, we developed a novel Y_1_Rs ligand APT-modified Mn-ZIF-90 nanosystem with both active-targeting and pH-responsive release to achieve excellent MRI contrast and chemotherapeutic efficacy of a breast tumor in vivo. The APT-Mn-ZIF-90 had higher drug loading than the ZIF-90 in the previous report. Further, this nanosystem could target both the cell membrane and the subcellular mitochondria of MCF-7 cells. Therefore, the DNA-damaging drugs loaded in this nanosystem could be released in the mitochondria, generating better therapeutic efficacy. The drug concentration of the APT-Mn-ZIF-90/5-Fu group was approximately two times higher than that of Mn-ZIF-90/5-Fu at 24 h post-injection. Especially, all tumors of the nude mice treated with APT-Mn-ZIF-90/5-Fu totally disappeared without recurrence, and no metastasis could be found in the liver. Active-targeting, high drug loading, and pH-responsive release were propitious to increase the drug concentration in the blood and more NP accumulation in the tumor, and fast drug release for killing tumor cells. Especially, this nanosystem itself could generate high resolution T_1_-weighted MR signals in the tumor site, which could be used to track the drug position in vivo. In addition, this nanosystem could be cleared out of the system within 14 days and showed no obvious toxicity and no damage to the mice. Thus, we believe this nanosystem could become a smart DDS in the future for more accurate and personalized imaging-guided therapy for breast cancer.

## Electronic supplementary material

Below is the link to the electronic supplementary material.

Supplementary material 1 (PDF 911 kb)
