# Supplementary material for: Manganese-Zeolitic Imidazolate Frameworks-90 with High Blood Circulation Stability for MRI-Guided Tumor Therapy
Source: Nanomicro Lett. 2019 Jul 23;11:61. doi: 10.1007/s40820-019-0292-y (PMC7770799; doi:10.1007/s40820-019-0292-y)
Supplement: Supplementary file 1 — Supplementary material 1 (PDF 911 kb) [file 40820_2019_292_MOESM1_ESM.pdf]

Supporting Information for

Manganese-Zeolitic Imidazolate Frameworks-90 with High Blood Circulation Stability for MRI Guided Tumor Therapy

Zhenqi Jiang[1, 3, †], Bo Yuan[1, †], Nianxiang Qiu[1], Yinjie Wang[1, 3], Li Sun[1, 3], Zhenni Wei[1], Yanyin Li[1, 3], Jianjun Zheng[2], Yinhua Jin[2], Yong Li[1], Shiyu Du[1], Juan Li[1, *], Aiguo Wu[1, *]

[1]Cixi Institute of Biomedical Engineering, CAS Key Laboratory of Magnetic Materials and Devices, & Key Laboratory of Additive Manufacturing Materials of Zhejiang Province, Ningbo Institute of Materials Technology and Engineering, Chinese Academy of Sciences, Ningbo 315201, People's Republic of China

[2]HwaMei Hospital, University of Chinese Academy of Sciences, Ningbo 315010, People's Republic of China

[3]University of Chinese Academy of Sciences, Beijing 100049, People's Republic of China

[†]Zhenqi Jiang and Bo Yuan contributed equally to this work.

*Corresponding authors. E-mail: aiguo@nimte.ac.cn (Aiguo Wu), lij@nimte.ac.cn (Juan Li)

S1 Experimental

S1.1 Materials and Regents

Zn $(CH_3COOH)_2 \cdot 2H_2O$ and imidazolate-2-carboxyaldehyde (2-ICA) were purchased from Infinity Scientific Co, Ltd (Beijing, China). Mn $(CH_3COOH)_2$, N, N-dimethylformamide (DMF), methanol (MeOH) were purchased from Aladdin Industrial Inc. (Shanghai, China). [Asn^{28}, Pro^{30}, Trp^{32}]-NPY (25-36) (abbreviation: APT) (sequence: Arg-His-Tyr-Asn-Asn-Pro-Ile-Trp-Arg-Gln-Arg-Tyr) was synthesized by the NJPeptide Co, Ltd (Nanjing, China). CCK-8 testing box was purchased from Medchem Express (USA). DMEM medium, fetal bovine serum (FBS), penicillin and streptomycin were purchased from HyClone (USA). All products received without any purify. MCF-7 cell line got from Cell Bank of the Chinese Academy of Sciences (Shanghai, China).

S1.2 Molecular Docking of APT to Y_1R

The 3D structural model of Y_1 receptor (Y_1R) was constructed as previously reported (PDB code: 5ZBH) [S1]. The structure of APT was derived from the NMR structure of neuropeptide Y (PDB code: 1RON) [S2], while the residues of neuropeptide Y were mutated to amino acid residues to get the structure of APT. Peptide docking of APT was completed using Rosetta's FlexPepDock application [S3]. The molecular dynamics simulation of APT to Y_1R was further evaluated by the GROMACS program [S4].

S1.3 Characterization of APT-Mn-ZIF-90/5-Fu

A particle size-zeta potential analyzer (Nano-ZS, Malvern, England) was used for the measurement of particle size and zeta potential of the APT-Mn-ZIF-90 in cell culture medium at room temperature. High-resolution transmission electron microscopy (HRTEM) images were recorded by a JEOL2100 (JEOL, Japan). The functional groups were investigated using fourier transform infrared spectroscopy (FT-IR) (Cary660 + 620, Agilent). The morphology was investigated through field emission scanning electron microscopy (FE-SEM) (S-4800, Hitachi), with a 10 kV acceleration voltage. The structural properties of samples were investigated by X-ray diffraction (XRD) using an X-ray powder diffractometer (XRD, D8 Discover, Bruker AXS). N_2 adsorption analysis was performed on the surface area and porosimetry instrument (ASAP 2020, Micromeritics) at 77 K. The concentration of Mn of samples was analyzed by ICP-OES (Optima 2100, Perkin-Elmer, USA).

S1.4 Magnetic Resonance Imaging (MRI) Test *in vitro*

The *in vitro* relaxation time (T_1 and T_2) of APT-Mn-ZIF-90 were measured on a 0.47 T Micro MR instrument at the frequency of 23.318 MHz (Niumag, Shanghai, China). The various Mn concentrations of APT-Mn-ZIF-90 samples in cell culture medium. Tw = 6000 ms. The slope of T_1 and T_2 were calculated by the value of longitudinal (r_1) and transverse (r_2) relativities against Mn concentrations. The T_1 MR images were acquired when TR = 200 ms, TE = 18.2 ms.

S1.5 Cellular Uptake of APT-Mn-ZIF-90 *in vitro*

Before laser scanning confocal microscope (LSCM) investigation, the MCF-7 cells were incubated with RhB loaded Mn-ZIF-90 or APT-Mn-ZIF-90 nanoparticles for 24 h. After 8 h incubation, 1 μM rhodamine 123 as a mitochondria tracker was added and incubated for 15 min, then the cells were fixed by tissue fixative. The fluorescent images were taken by LSCM (EX 488nm, EM500-545 and 560-620 nm) (TCS SP5 II, Leica, Germany). To quantify the cellular uptake difference, the MCF-7 cells were treated with Mn-ZIF-90/RhB or APT-Mn-ZIF-90/RhB (RhB: 20 μg mL^{-1}). The cells were removed from six-well plate after 8 h incubation and resuspended in PBS. Every 10000 cells were analysis by using flow cytometer analysis (FACSCalibur, BD, USA),

and the mean fluorescence intensity (MFI) of cells were calculated for comparison.

For the investigation of elements uptake in MCF-7 cells, X-ray fluorescence microscopy at Shanghai Synchrotron Radiation Facility (SSRF, Shanghai, China) was used as our group pervious reported [S5]. In general, the cells were grown on sterile Malay films for 24 h, and then were cultured with Mn-ZIF-90 and APT-Mn-ZIF-90 (100 μg mL^{-1}) for 8 h. After fixing by tissue fixative and washed with pure water, the cells were tested. The hard X-rays BL15U beamline at SSR was also used to get distribution mapping of elements (Cl, Zn and Mn) at the condition of energy of the X-ray was 10 keV with the beam spot was 0.5×0.5 μm $(step*s)^{-1}$.

For the investigation of nanoparticles uptake in MCF-7 cells, soft X-ray fiber microscopy at University of Science and Technology of China (Hefei, China). Briefly, MCF-7 were incubated in 6-well-culture plates for 24 h at 37 °C with 5% CO_2, then the medium was replaced by APT-Mn-ZIF-90 (100 μg mL^{-1}) for 4 h. The cells were dissociated by 0.25% trypsin and centrifuged then dispersed in PBS. The solution was dropped on a nickel mesh. 3-D cell images were obtained at Soft X Suspected Microscopic Imaging Beam Station.

To quantitatively measure the content of Mn in MCF-7 cells, the cells were incubated with different concentration of Mn-ZIF-90 and APT-Mn-ZIF-90 solution for 4 h. After then, cells were collected and re-suspended in PBS (1×10^5 cells/ml) for ICP-OES (Optima 2100, Perkin-Elmer, USA).

S1.6 Cell Cytotoxicity Assays

10000 MCF-7 cells were seeded in a 96-well-culture plates for 24 h at 37 °C with 5% CO_2. Then the cells were treated with different concentration of APT-Mn-ZIF-90 or free 5-Fu, Mn-ZIF-90/5-Fu and APT-Mn-ZIF-90/5-Fu in the 5-Fu concentration. After another 24 h, adding CCK-8 solution (10 μL) and for another 2 h incubation at 37 °C. The absorbance intensity every well was used by an automated plate reader (iMark (168-1130), Biorad, U.S.) at 450 nm. The data was processed according to our pervious reported [S6].

S2 Supplementary Figures and Tables

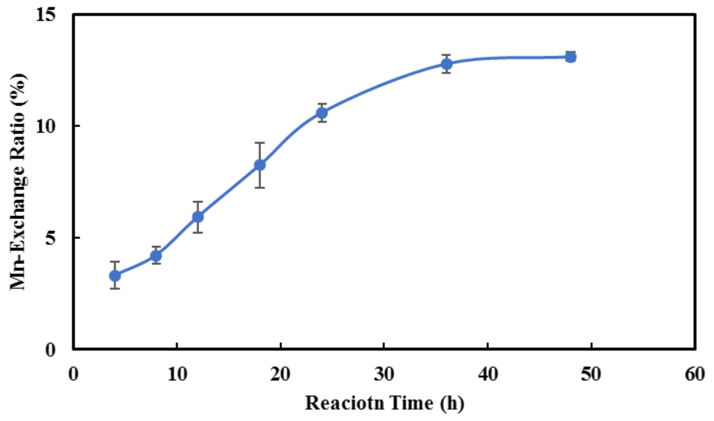

Fig. S1 Mn-exchange ratio under different reaction time. The molar ratio of Mn^{2+} to ZIF-90 was 3, and the temperature was at 55 °C

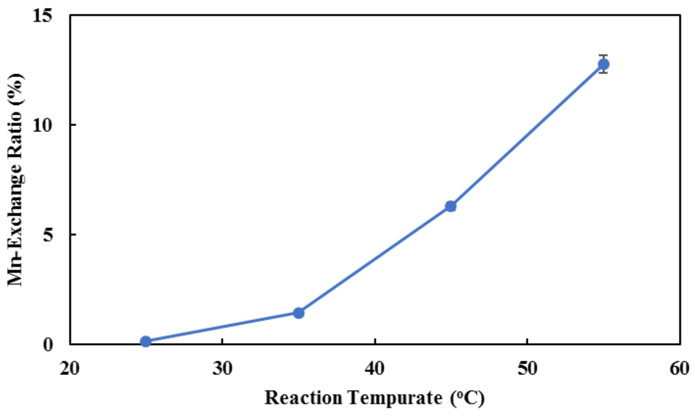

Fig. S2 Mn-exchange ratio for different reaction temperate. The molar ratio of $Mn^{2+}$to ZIF-90 was 3, and the reaction time was 36 h

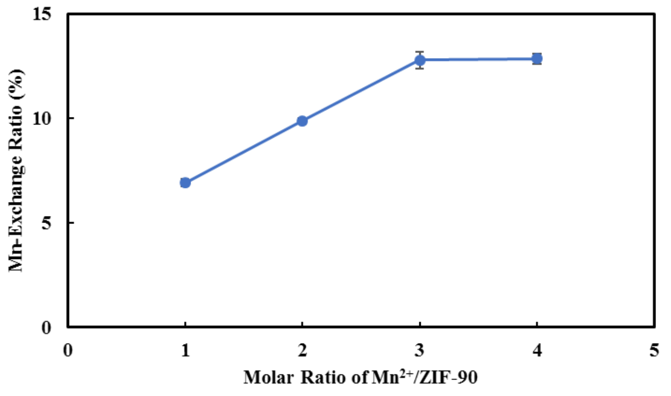

Fig. S3 Mn-exchange ratio for different molar ratio of Mn^{2+} to ZIF-90. The reaction temperature was at 55 °C, and the reaction time was 36 h

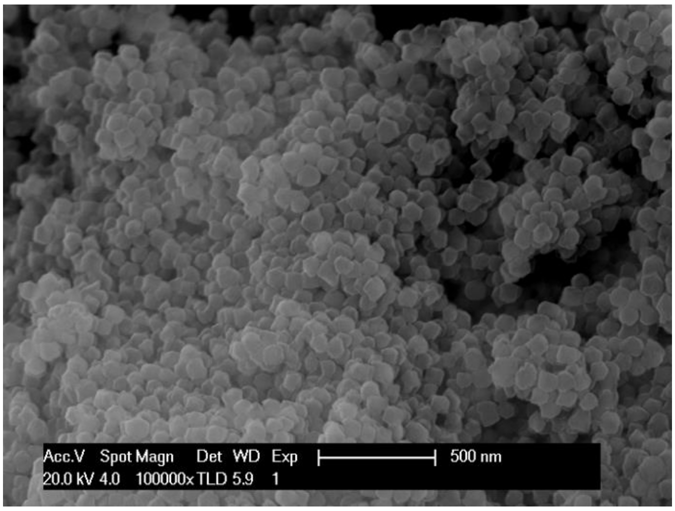

Fig. S4 SEM imaging of ZIF-90

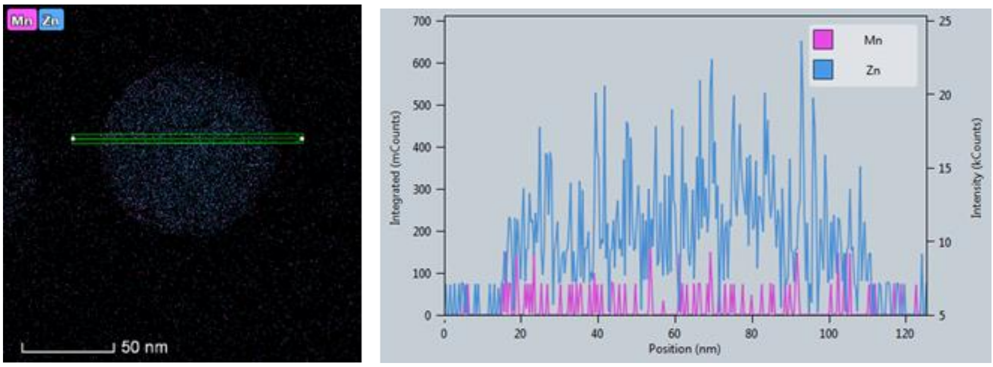

Fig. S5 Merge image of Zn and Mn and their element intensity

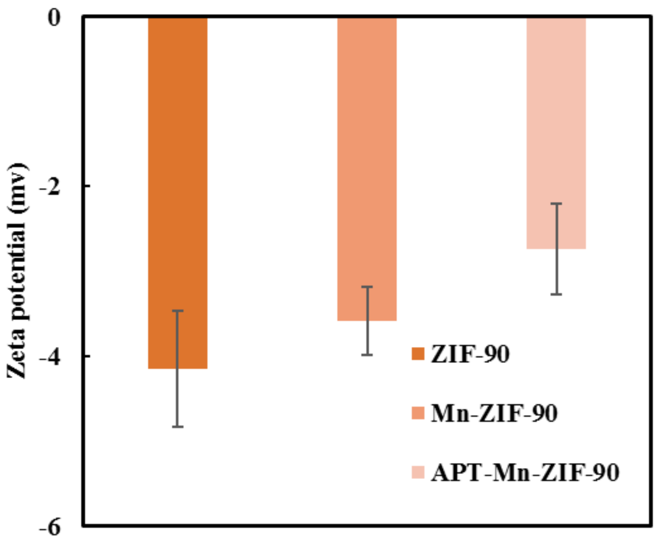

Fig. S6 Zeta-potential of ZIF-90, Mn-ZIF-90, and APT-Mn-ZIF-90

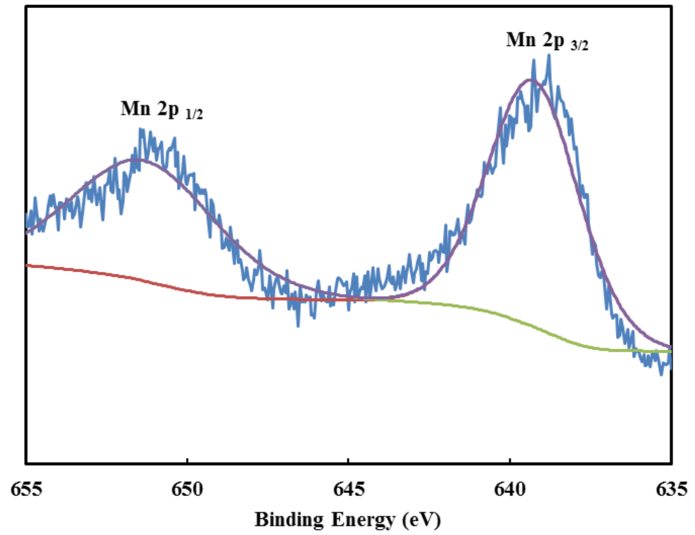

Fig. S7 Mn 2p XPS spectra of Mn-ZIF-90

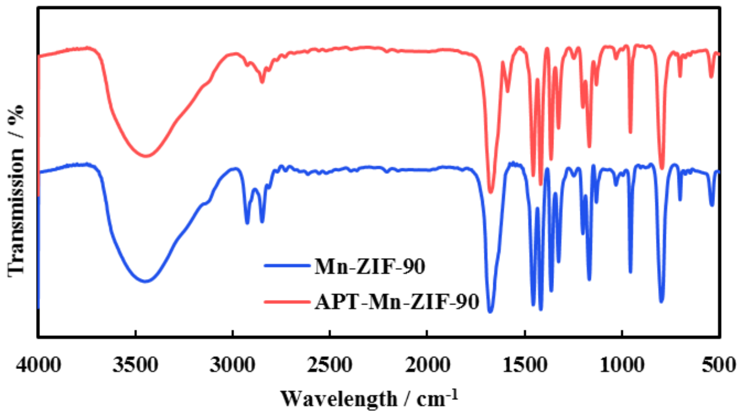

Fig. S8 FT-IR of Mn-ZIF-90 and APT-Mn-ZIF-90

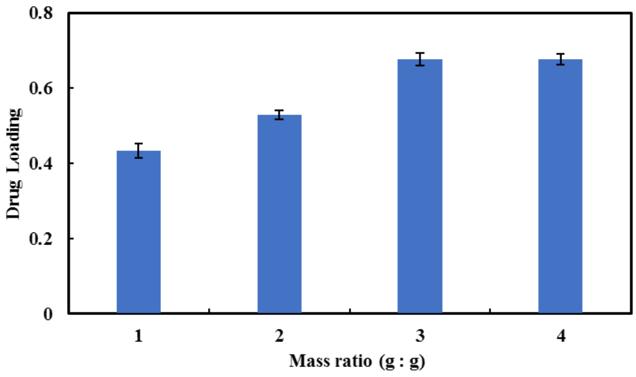

Fig. S9 Drug loading of APT-Mn-ZIF-90 at different mass ratio of 5-Fu to APT-Mn-ZIF-90

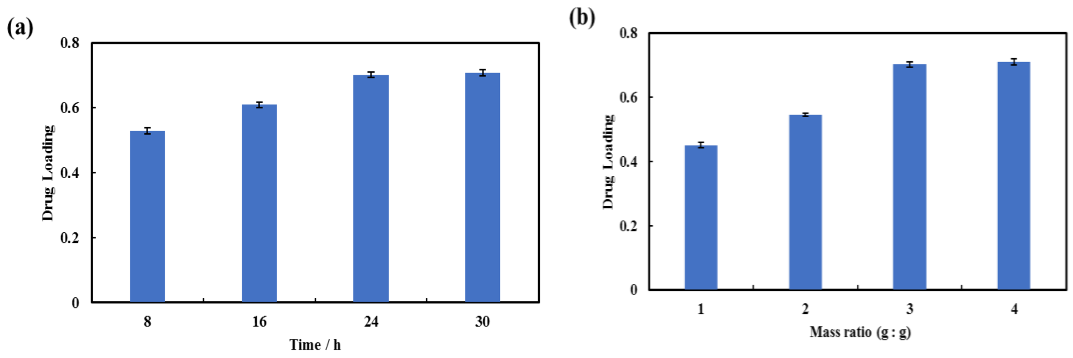

Fig. S10 Drug loading of Mn-ZIF-90 at different **a** stirring time and **b** mass ratio of 5-Fu to Mn-ZIF-90

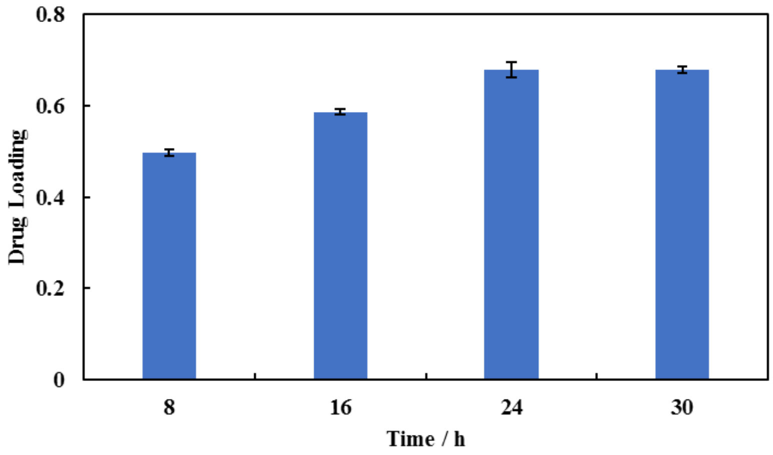

Fig. S11 Drug loading of ZIF-90 at different stirring time

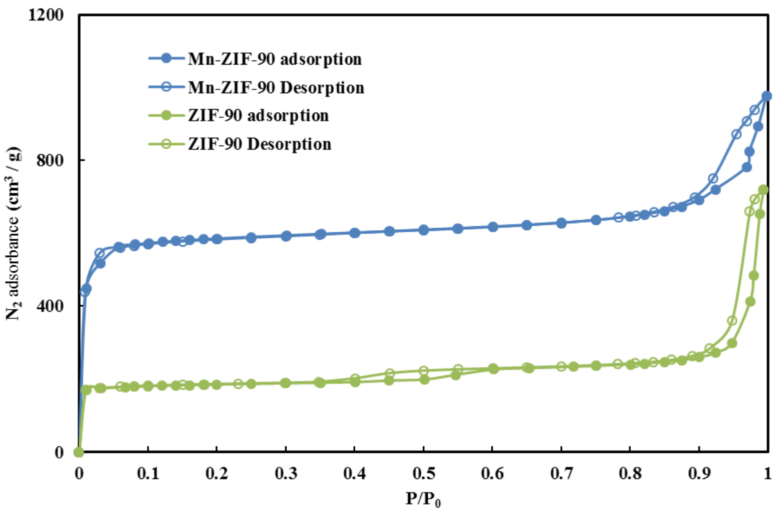

Fig. S12 N_2 absorbance of ZIF-90 and Mn-ZIF-90

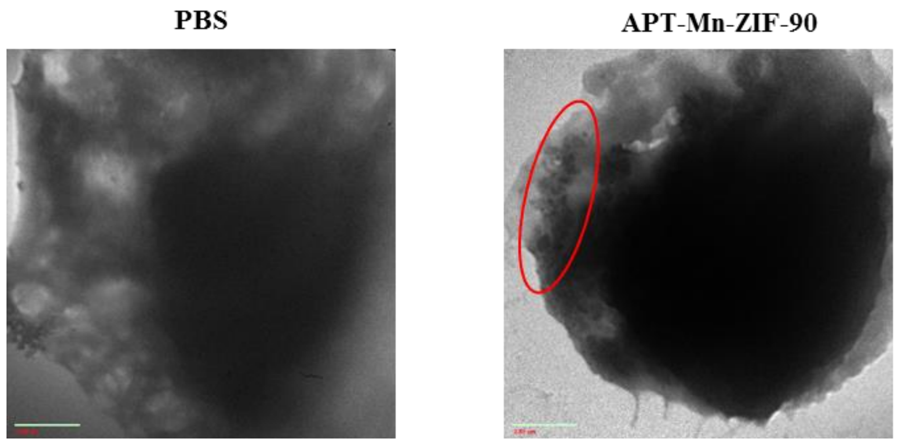

Fig. S13 XFM images of cells treated by PBS and APT-Mn-ZIF-90 after 8 h

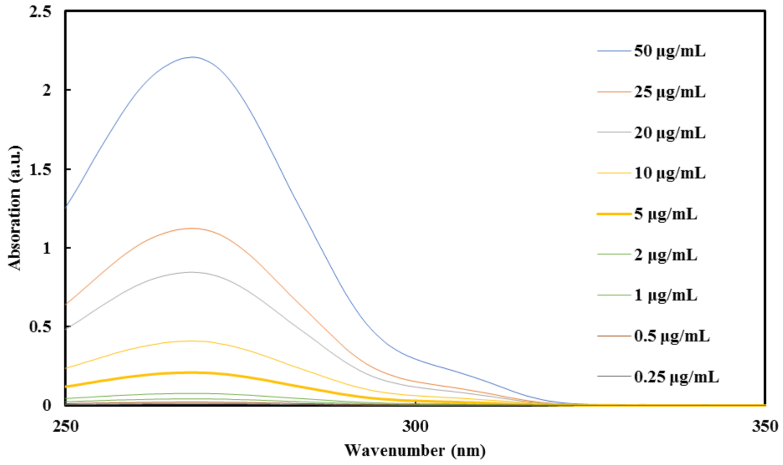

Fig. S14 UV-Vis spectra of 5-Fu at different concentration

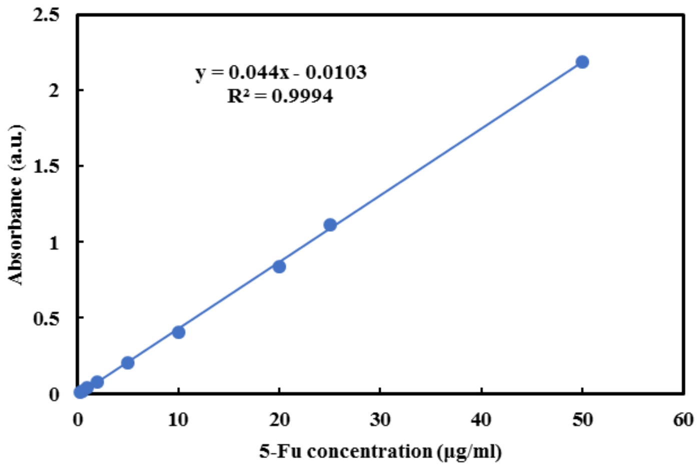

Fig. S15 Standard curve of 5-Fu from UV-Vis spectra

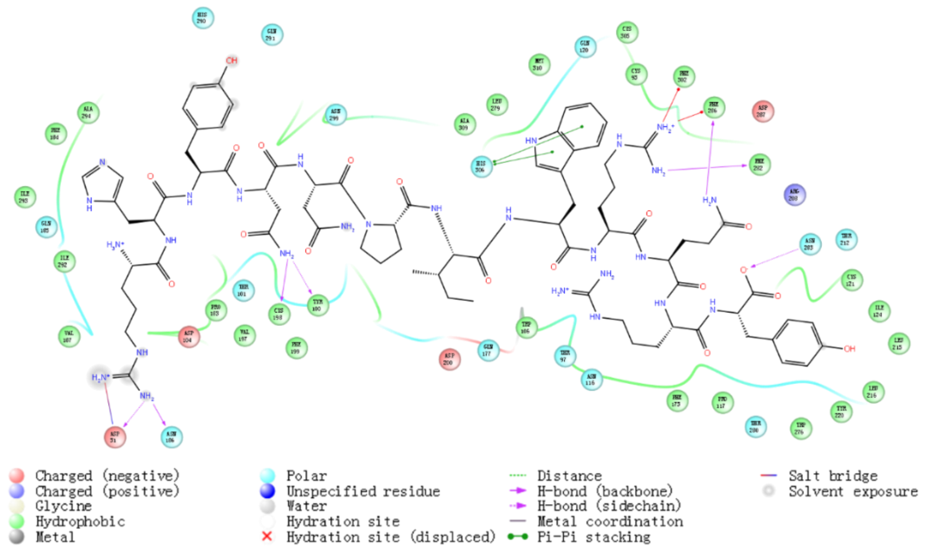

Fig. S16 Schematic representation of the interactions between APT and Y_1R. The residue of the Y_1R was represented by spheres

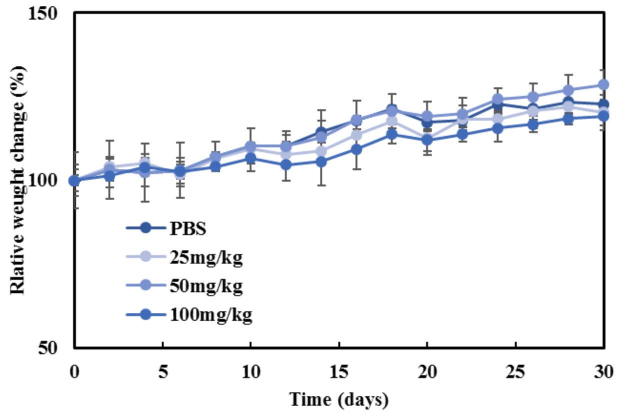

Fig. S17 The relative weight change of mice after i.v. injection of different dosage of APT-Mn-ZIF-90 after 30 days

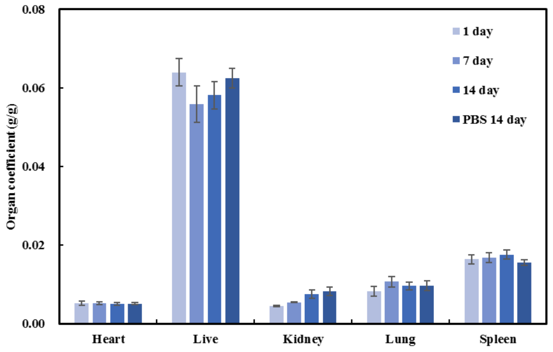

Fig. S18 Organ coefficients of Balb/C mice after i.v. injection of APT-Mn-ZIF-90 (50 mg kg^{-1}) at different time

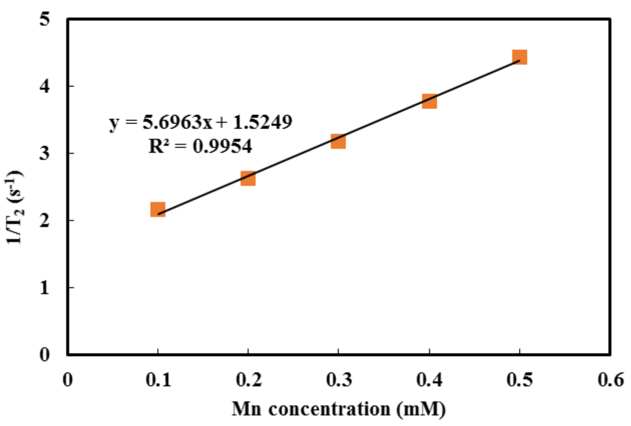

Fig. S19 The slope of r_2 value of APT-Mn-ZIF-90 at different concentration of Mn^{2+}

Table S1 Mn exchange ratio and yield of Mn-ZIF-90 at different condition

Sample No.	Reaction Time (h)	Reaction Temperate (°C)	Molar ratio of Mn^{2+} to ZIF-90	Mn Exchange Ratio (%)	Yield (%)
**1**	4	55	3	3.32 ± 0.62	87.19 ± 1.89
**2**	8	55	3	4.21 ± 0.38	83.53 ± 3.16
**3**	12	55	3	5.91 ± 0.70	81.73 ± 4.28
**4**	18	55	3	8.24 ± 1.02	75.89 ± 3.49
**5**	24	55	3	10.59 ± 0.42	72.54 ± 4.15
**6**	36	55	3	12.78 ± 0.39	61.17 ± 3.14
**7**	48	55	3	13.10 ± 0.20	44.42 ± 2.41
**8**	36	25	3	0.14 ± 0.03	91.56 ± 2.49
**9**	36	35	3	1.44 ± 0.05	73.16 ± 4.12
**10**	36	45	3	6.30 ± 0.17	69.12 ± 1.28
**11**	36	65	3	N.A.	N.A.[a]
**12**	36	55	1	6.91 ± 0.18	72.46 ± 1.17
**13**	36	55	2	9.89 ± 0.13	70.13 ± 0.89
**14**	36	55	4	12.86 ± 0.24	54.12 ± 1.89

[a] N.A. No product was found (n=3)

Table S2 Survival ratio of different nanoparticles at different given dosage

Nanoparticles	12.5 mg kg^{-1}	25 mg kg^{-1}	35 mg kg^{-1}	50 mg kg^{-1}	75 mg kg^{-1}	100 mg kg^{-1}
**ZIF-90**	5/5	5/5	5/5	5/5	5/5	5/5
**Mn-ZIF-90**	5/5	5/5	5/5	5/5	5/5	5/5
**APT-Mn-ZIF-90**	5/5	5/5	5/5	5/5	5/5	5/5

Supplementary References

[S1] Z. Yang, S. Han, M. Keller, A. Kaiser, B.J. Bender et al., Structural basis of ligand binding modes at the neuropeptide Y Y1 receptor. Nature **556**(7702), 520-524 (2018). https://doi.org/10.1038/s41586-018-0046-x

[S2] S.A. Monks, G. Karagianis, G.J. Howlett, R.S. Norton, Solution structure of human neuropeptide Y. J. Biomol. NMR **8**(4), 379-390 (1996). https://doi.org/10.1007/bf00228141

[S3] B. Raveh, N. London, O. Schueler-Furman, Sub-angstrom modeling of complexes between flexible peptides and globular proteins. Proteins: Struct. Funct. Bioinf. **78**(9), 2029-2040 (2010). https://doi.org/10.1002/prot.22716

[S4] M.J. Abraham, T. Murtola, R. Schulz, S. Páll, J.C. Smith, B. Hess, E. Lindahl, Gromacs: High performance molecular simulations through multi-level parallelism from laptops to supercomputers. SoftwareX **1-2**, 19-25 (2015). https://doi.org/10.1016/j.softx.2015.06.001

[S5] S. Wang, W. Ren, J. Wang, Z. Jiang, M. Saeed, L. Zhang, A. Li, A. Wu, Black TiO_2-based nanoprobes for T1-weighted MRI-guided photothermal therapy in CD133 high expressed pancreatic cancer stem-like cells. Biomater. Sci. **6**(8), 2209-2218 (2018). https://doi.org/10.1039/C8BM00454D

[S6] J. Li, Z. Shen, X. Ma, W. Ren, L. Xiang, A. Gong, T. Xia, J. Guo, A. Wu, Neuropeptide Y Y1 receptors meditate targeted delivery of anticancer drug with encapsulated nanoparticles to breast cancer cells with high selectivity and its potential for breast cancer therapy. ACS Appl. Mater. Interfaces **7**(9), 5574-5582 (2015). https://doi.org/10.1021/acsami.5b00270
